# Supplementary material for: Analysis of Patient Safety Incidents in Primary Care Reported in an Electronic Registry Application
Source: Int J Environ Res Public Health. 2021 Aug 25;18(17):8941. doi: 10.3390/ijerph18178941 (PMC8430626; doi:10.3390/ijerph18178941)
Supplement: Supplementary file 1 [file ijerph-18-08941-s001.zip › ijerph-1310594-supplementary.pdf]

Supplementary material

Analysis of patient safety incidents in primary care reported in an electronic registry application.

Montserrat Gens-Barberà, Núria Hernández-Vidal, Elisa Vidal-Esteve, Yolanda Mengíbar-García, Inmaculada Hospital-Guardiola, Eva María Oya-Girona, Ferran Bejarano-Romero, Carles Castro-Muniain, Eva Satué-Gracia, Cristina Rey-Reñones, Francisco Martín-Luján.

This appendix has been provided by the authors to give readers additional information about their work.

Supplementary material S1. Description of variables in the notification registry of patient safety incidents. Platform for Patient Safety Management with the TPSC Cloud™ (Tarragona Regional Management of the Catalan Institute of Health)

Type of Primary Care Unit /Centre

1 - Primary Care Health Centre
2 - Primary care emergency centre (CUAP, in its Catalan acronym) and
3 - Sexual and reproductive health services (ASSIR, in its Catalan acronym)

Professional notifier

1 - Physicians: family and community medicine, and paediatrics
2 - Nursing
3 - Nursing assistant
4 - Health technician
5 - Admin officer
6 - Health assistant and orderly
7 - Others (midwife, dentist, psychologist, physiotherapist ...)

Type of incident

World Health Organisation (WHO) - 10 categories:

1 - Falls and other accidents
2 - Patient behaviour
3 - Clinical equipment and devices
4 - Analog and digital documentation
5 - Clinical management and procedures
6 - Clinical-administrative management
7 - Infection associated with health care
8 - Severe nosocomial pressure ulcers
9 - Infrastructures and facilities
10 - Medication

Catalan Health Department Accreditation Model (HDAM) - 15 categories:

1 - Emergency Care
2 - Continuity of care
3 - Health education
4 - Ethics and rights of citizens
5 - Management of clinical material
6 - Waste management
7 - Laboratory
8 - Healthcare process
9 - Administration
10 - Diagnostic Imaging
11 - General services (cleaning, security, infrastructures ...)
12 - Social work
13 - Safe use of medicines
14 - Vaccines
15 - Infection surveillance, prevention and control

**Severity of Incident (WHO criteria)**
a) Incident that does not reach the patient<br>- Notifiable circumstance that may cause error<br>- Near-miss incident before reaching the patient<br>b) Incident that reaches the patient without damage or injury<br>c) Incident with damage or adverse event causing:<br>- mild damage: no harmful, but requires minimal observation or intervention.<br>- moderate damage: requires treatment and / or has caused temporary injury.<br>- severe damage: has required intensive treatment or resulted in permanent damage.<br>- extreme damage: has caused a near death injury and / or has caused or contributed to death.
**Risk Matrix** (classified based on)
**a) Risk**<br>- Very low (verification of possible presentation trends in the health centre).<br>- Low (verification of possible presentation trends in the affected area / service).<br>- Moderate (requires verification and monitoring of possible presentation trends in the affected area / service).<br>- High (requires analysis of the incident and adoption of corrective measures in the affected area / service).<br>- Extreme (requires detailed analysis and immediate corrective action).<br>**b) Probability that it will happen again**<br>- Very rare (only in exceptional circumstances, often> 5 years).<br>- Uncommon (sometime, every 2-5 years).<br>- Possible / occasional (sometime, 1-2 times per year).<br>- Probable (under some circumstances, several times per year).<br>- Frequent (expected to occur in the next few weeks or months).
**Causal factors according to the APEAS study**
Classified into five categories and 36 subcategories related to:<br>- Communication (12): doctor-patient, doctor-doctor, doctor-nurse, doctor-admin officer, nurse-patient, nurse-nurse, nurse-admin officer, admin officer-patient, admin officer-admin officer, cultural barrier, language barrier, another communication factor.<br>- Management (5): long waiting list, erroneous citation, problems with the EHR, mistake in health information, error in patient identification.<br>- Care delivery (4): inadequate management of patient, inadequate technique, inadequate management of warning signs, inadequate management of procedure.<br>- Diagnosis (4): delay in diagnosis, diagnostic error, referral to specialist delayed and other causes.<br>- Medication (11): wrong dose, lack of adherence, missed dose, wrong medication, drug interaction, wrong administration frequency, wrong treatment duration, wrong patient, ineffective prescription, insufficient monitoring, preparation or handling error.
**Contributing factors**
Classified in 5 categories related to:<br>- Environment<br>- External<br>- Organisation<br>- Patient<br>- Professional

**Preventability**
- Potentially preventable<br>- Unclear<br>- Not preventable
**Resolution level**
- Primary Care Centre<br>- Central Patient Safety Functional Unit<br>- Primary Care Clinical Management<br>- Transversal Patient Safety Functional Unit with referral hospitals
**Safe practices and improvement actions**
The analysis and management of reported PS incidents lead to the implementation of improvement actions and safe practices:<br>- Improvement team<br>- Teaching<br>- Report review<br>- Committee / management
**Quality of notification**
- Correct / Divergent<br>The experts at the Central Safety Functional Unit of the Primary Care Management verify that the incident meets all notification requirements (all sections are correctly filled in). If they detect a discrepancy in the information provided, the notification is classified as "discrepant" and the experts advise the patient safety professional of the centre where the incident happened, who is expected to clarify any issues.
**Status**
- Solved<br>- Pending regional Patient Safety Functional Unit<br>- Pending Primary Care Management<br>- Pending other

Supplementary material S2. Table S2a and S2b show the matrix of analyses of contributing factors for PS incidents and adverse events, respectively, according to the WHO classification.

Table S2a. Matrix of contributing factors (Total incidents; n=1129)

	Professional	Patient	Organisation	Environment	External
**Professional**	350 (31.0%)	32 (2.8%)	197 (17.4%)	8 (0.7%)	43 (3.8%)
**Patient**		9 (0.8%)	9 (0.8%)	2 (0.2%)	2 (0.2%)
**Organisation**			170 (15.1%)	9 (0.8%)	30 (2.7%)
**Environment**				12 (1.1%)	3 (0.3%)
**External**					215 (19.0%)

Table S2b. Matrix of contributing factors (Adverse events, n=96)

	Professional	Patient	Organisation	Environment	External
**Professional**	22 (22.9%)	5 (5.2%)	12 (12.5)	0 (0.0%)	7 (7.3%)
**Patient**		2 (2.1%)	0 (0.0%)	1 (1.0%)	0 (0.0%)
**Organisation**			18 (18.8)	3 (3.1%)	6 (6.3%)
**Environment**				1 (1.0%)	0 (0.0%)
**External**					19 (19.8%)
